# Image-Guided Local Treatment for Unresectable Intrahepatic Cholangiocarcinoma—Role of Interventional Radiology

**DOI:** 10.3390/jcm10235574

**Published:** 2021-11-26

**Authors:** Matthias P. Fabritius, Najib Ben Khaled, Wolfgang G. Kunz, Jens Ricke, Max Seidensticker

**Affiliations:** 1 Department of Radiology, University Hospital, LMU Munich, Marchioninistr. 15, 81377 Munich, Germany; wolfgang.kunz@med.uni-muenchen.de (W.G.K.); jens.ricke@med.uni-muenchen.de (J.R.); 2 Department of Medicine II, University Hospital, LMU Munich, Marchioninistr. 15, 81377 Munich, Germany; najib.benkhaled@med.uni-muenchen.de; 3 German Cancer Consortium (DKTK), Partner Site Munich, Marchioninistr. 15, 81377 Munich, Germany

**Keywords:** intrahepatic cholangiocarcinoma, interventional treatment, locoregional therapy, ablative therapy, TACE, radioembolization

## Abstract

Intrahepatic cholangiocarcinoma is a highly aggressive malignancy with an increasing incidence in recent years. Prognosis is poor and most patients are not eligible for resection at the time of initial diagnosis due to the anatomic location, inadequate hepatic reserve, limiting comorbidities or metastatic disease. Several locoregional therapies from the field of interventional radiology exist for patients who are not amenable for surgery, or in case of local recurrence as a single treatment modality or combined with systemic treatment. To date, evidence is limited, with most conclusions drawn from single-center studies with small patient cohorts, often treated in the salvage situation or for local recurrence after initial resection. Nevertheless, the results are promising and suggest a survival benefit in selected patients. This narrative review focuses on the use of different locoregional treatment options for intrahepatic cholangiocarcinoma.

## 1. Introduction

Intrahepatic cholangiocarcinoma (iCCA) is the second most common primary liver malignancy after hepatocellular carcinoma, and incidence and mortality have increased globally in the last decade [1,2,3,4]. iCCA has a poor prognosis and is often asymptomatic in early stages of the disease. It manifests as an incidental finding on imaging or, in advanced stages, with clinical symptoms such as abdominal pain, weight loss, nausea, and jaundice. Only about 20% of all patients are candidates for surgical resection, the only potentially curative treatment option at initial diagnosis due to its anatomic location, inadequate hepatic reserve, limiting comorbidities or metastatic disease [5]. The median survival for patients with untreated iCCA has been reported with 3–6 months [3,6]. Systemic chemotherapy with gemcitabine and cisplatin (gem/cis) improved the outcome, with a reported median overall survival (OS) of around 15 months [7]. Nevertheless, many patients have a chemo-refractory course or discontinue therapy due to associated severe side effects. To date, there is no consensus on the optimal treatment regime of unresectable, recurrent, and chemo-refractory iCCA, albeit several studies are currently investigating further approaches [8,9,10]. Targeted drugs showed promising outcomes in several studies and are now established as second-line therapies [11,12,13,14,15,16]. However, targetable mutations occur in only about half of patients [17,18,19]. Additionally, there are various image-guided local treatment options from the field of interventional radiology, but up until now the evidence has been low, with most conclusions drawn from retrospective single-center analyses. Nevertheless, the results are promising and suggest a survival benefit in the treatment of unresectable or recurrent iCCA in selected patients, even in an advanced, metastatic stage [20]. In addition, they serve the main goals of palliative therapy, namely to control local tumor growth and thus alleviate tumor-related symptoms, as well as improve and maintain quality of life.

## 2. Image-Guided Local Treatment Techniques

Various interventional radiologic techniques exist and have been shown to be safe and effective in treating liver metastases of different entities and primary liver tumors, with varying degrees of evidence regarding iCCA. Both the precise consideration of the respective treatment modality and patient selection may have a major impact on the success of the treatment, as well as on patient safety. 

In general, a distinction is made between local ablative and locoregional procedures. Local ablative techniques such as microwave ablation (MWA), radiofrequency ablation (RFA), or image-guided interstitial high-dose-rate brachytherapy (HDR-BT), a catheter-based radiotherapy, aim at total and permanent local tumor destruction and, thus, basically allow complete tumor ablation with, if successful, excellent local tumor control. In contrast, locoregional techniques such as trans-arterial chemoembolization (TACE) and radioembolization (RE or TARE), often achieve only partial remission. Therefore, local ablation is generally the preferred approach when feasible. The choice of technique (local ablation or locoregional treatment) is mainly influenced by the number, size, and location of the tumor(s). Based on our own experiences and the official standard of practice of the Cardiovascular and Interventional Radiological Society of Europe (CIRSE), thermal-based ablative techniques such as RFA and MWA are more suitable for the treatment of fewer (<3, possibly up to 5) and smaller tumors that are not close to the hepatic hilum or surrounding organs [20,21]. Adjacent large blood vessels cause cooling effects that may counteract adequate heat generation and prevent complete tumor ablation [22,23]. Proximity to the hepatic hilum carries the risk of damage to the heat-sensitive central bile ducts, and a peripheral location with proximity to heat-sensitive organs (gallbladder, stomach, colon, duodenum, heart) carries the risk of organ damage/perforation [24,25,26]. Regarding size limitations, a maximum tumor diameter of 5 cm for singular lesions and 3.5 cm for multifocal metastases is recommended by the German Society for Interventional Radiology and Minimally Invasive Therapy (DEGIR) to ensure safe ablation [27]. Since iCCA patients are usually diagnosed at an advanced stage, these limitations often exclude the use of thermal procedures so they play a minor role in the therapeutic regimen, mainly in circumscribed recurrences after initial resection. In contrast, interstitial HDR-BT is largely independent of tumor size and location and can therefore be used for larger tumors as well as for lesions near hilar structures [28]. It has been used for the treatment of different hepatic malignancies, and promising results have been presented, especially in colorectal cancer metastases and hepatocellular carcinoma, as well as in iCCA [20,29,30,31]. Briefly, a high-dose-rate iridium-192 source is placed in the tumor per afterloading technique, after percutaneous image-guided positioning of special, hollow brachytherapy catheters (CT, MRI or ultrasound), providing a prescribed minimum dose of 20 Gy for the clinical target volume [29,32,33,34,35]. For patients with multifocal or diffuse liver involvement, locoregional trans-arterial procedures (TACE or RE) are available as alternative treatment options if good performance status is maintained and liver function is adequate. The rationale of trans-arterial therapies relies mainly on the dual blood supply of the liver and the difference in the blood supply between liver tumors and the liver parenchyma. In contrast to the liver tissue, which is mainly fed by the portal venous system, liver tumors, in special hypervascularized ones, are mainly dependent on the hepatic artery for their oxygen and nutrient supply [36]. The administration of the treatment agent via the hepatic artery results in an accumulation and high local concentration within the tumor with generally only a little effect on normal liver tissue.

## 3. Local Ablative Treatment in iCCA

### 3.1. Thermal Ablation (RFA/MWA)

As mentioned above, thermal ablation plays a minor role in the treatment of iCCA as they are usually diagnosed in an advanced stage, with tumor size and tumor distribution beyond accessibility to local ablative treatments, especially for thermal ablations. A recent analysis of patients with small (≤5 cm), early-stage, primary iCCA who were screened from the Surveillance, Epidemiology, and End Results (SEER) database—a public database that collects survival and incidence data of various types of cancers, and covers more than 25% of the United States’ population—indicated that surgical resection is superior to RFA as a first-line treatment [37]. However, small tumors (preferably <3 cm), as primary or as a recurrence after the initial surgery, in patients who are not willing or eligible for re-resection are reasonable candidates for thermal ablation according to the latest systematic meta-analysis [38].

Special awareness should be directed to possible complications due to the presence of a hepaticojejunostomy or compromised hepatopancreatic ampulla, as abscess formation after thermal ablation is reported to develop in up to 2% of patients, mostly in consequence of colonizing enteric bacteria [39,40]. Treatment of these abscesses can be cumbersome since the environment is ischemic, and relapse is frequent due to lasting contamination via the biliary tree. Another dreaded complication of thermal ablation is direct damage to the bile ducts adjacent to the ablation zone, especially if the ablation zone is in proximity to the liver hilum/central bile ducts; bile duct necrosis can lead to severe complications with bilomas and/or stenosis [26].

Kim et al. reported good results for 13 patients undergoing primary RFA of histologically proven iCCA who were not amenable to curative surgery [41]. Patients had three or fewer tumors, no imaging evidence of vascular invasion and mainly no extrahepatic disease, except one patient with bone metastases. Local control was successful in 88% at a median follow-up of 19.5 months. The median overall survival (OS) was 38.5 months. In the two treatment failures, the tumors were more than 5 cm in diameter. One out of 17 interventions was complicated by a liver abscess. Further, mainly small retrospective single-center studies confirmed these good results, both as primary therapy and in local recurrences with median OS up to 60 months after local treatment and low major complication rates (mean < 5%) [42,43,44,45,46,47,48,49,50,51,52,53]. Remarkably, in a study of patients with iCCA recurrence after primary surgical resection, Zhang et al. found that major complications were significantly less frequent after local ablation (RFA and MWA) compared to surgical re-resection (3.9% vs. 46.9%) with the same median OS (21.3 vs. 20.3 months) [54]. In principle, thermal ablative procedures appear to be safe in iCCA as Díaz-Gonzàles was able to demonstrate comparable results in a cohort of patients with liver cirrhosis [55]. An important prognostic factor in all studies was complete ablation coverage of the tumor.

### 3.2. Image-Guided Interstitial High-Dose-Rate Brachytherapy (HDR-BT)

Interstitial HDR-BT is generally independent of tumor size and location and can therefore be deployed in selected patients with iCCA in whom MWA or RFA is not feasible. As with thermal ablation, there is a risk of abscess formation, especially in the presence of a hepaticojejunostomy or an impaired ampulla [56,57]. However, unlike thermal ablation, the treatment limitations in terms of potential bile duct complications are lower. Dosimetric planning for BT allows the exact measurement of potential exposure, and the adjustment of dosimetry if exposure is too high. A threshold of approximately 20 Gy is reported for the development of bile duct complications [58].

In a study by Schnapauff et al., 15 therapy-naïve patients with unresectable iCCA and limited disease (<5 hepatic lesions, maximum size of 12 cm) who received repeated HDR-BT achieved a median OS of 14 months after treatment and 21 months after initial diagnosis, respectively [31]. It is important to notice that a certain number of patients in this study received systemic chemotherapy in the course after HDR-BT, which certainly limits the interpretation of the results. Moreover, effective treatment of iCCA recurrences with HDR-BT after initial resection was shown by Kamphues et al. in a case series of 10 patients with 1-year and 5-year survival rates of 77.1% and 51.4%, respectively [59]. Jonczyk et al. confirmed that HDR-BT is also sufficient in larger iCCA (≥4 cm) if full coverage with therapeutic doses is achieved [32]. Structured data on combined local ablation and systemic therapy have not yet been published. However, a trial evaluating the efficacy of HDR-BT in combination with gem/cis in unresectable iCCA patients is currently recruiting (DRKS00007161). Altogether, HDR-BT is a promising and safe technique for patients who are not eligible for tumor resection.

## 4. Trans-Arterial Locoregional Treatment in iCCA

One of the major challenges is the treatment of patients who have maintained a good performance status, but present with extensive liver involvement—beyond or alongside systemic chemotherapy. Since iCCA is known to have only a limited response to systemic chemotherapy, intra-arterial therapies are frequently attempted, most commonly TACE and RE, although there is no evidence of their superiority. Previous analyses suggest that they provide suboptimal overall objective response rates with good safety profiles [60,61,62]. However, they achieve high local response rates, which are probably a better descriptor of efficacy and more important for prognosis. When interpreting the published data, it is important to note that patients undergoing such procedures are often in an advanced and chemotherapy-refractory stage of the disease [63]. Overall, a selection bias must be assumed, as these patients are strongly preselected due to various reasons, such as general health status and comorbidities, stage of the disease including tumor burden/number and location of lesions, and lack of therapeutic alternatives, which in turn have a considerable influence on survival [64].

### 4.1. TACE

Several retrospective studies analyzed the outcome after TACE in iCCA patients [65,66,67,68,69,70,71,72,73]. Unfortunately, the TACE procedure is not standardized, so that even a comparison of the studies among each other limits a conclusion. Various chemotherapeutic drugs in various concentrations were used (such as doxorubicin, cisplatin, mitomycin, gemcitabine and epirubicin) and administered, mixed with ethiodized oil (so-called conventional TACE) or loaded on embolic beads (so-called drug-eluting beads TACE) [72,74]. Pooled median OS in a systematic meta-analysis was 14.2 months, and the objective response rate mostly did not exceed 25% [63]. The most frequent positive prognostic factors for the outcome after TACE were good liver function, objective tumor response and low tumor extent [65,66,67,68,69,70,71,72,73]. Different to the liver parenchyma, the biliary tree is supplied by the artery only. Thus, arterial embolization can induce ischemia to the biliary tree leading to necrosis and bilomas with a risk of superinfection. Risk for biliary tree ischemia is higher in non-cirrhotic patients (since cirrhotic patients compensate arterial ischemia via shunts from the portal tract) and reaches a frequency of up to 30% [75].

### 4.2. Radioembolization

RE in iCCA patients was analyzed in several retrospective studies in different clinical settings: unresectable therapy-naïve and recurrent patients, in combination with systemic therapy, and in the chemotherapy-refractory stage [64,76,77,78,79,80,81,82,83,84,85,86,87,88,89,90,91,92,93,94]. Pooled median OS after RE was 13.2 months, comparable to OS after TACE [63]. However, it should be noted that the patients who received RE were often in a more advanced stage of the disease and, due to the heterogeneity of the study cohorts and the respective time of RE-application in the course of the disease, these data should be interpreted carefully. The exact role in the therapy algorithm is still a matter of debate. Previous studies evaluating the combination of RE and chemotherapy with gem/cis in unresectable or relapsed patients yielded variable results, with no clear superiority of combined treatment compared with RE alone [76,95]. Importantly, no study indicated an increase in liver toxicity when both treatments were combined. In a prospective Phase 2 study, RE in combination with gem/cis as the first-line treatment was associated with an increased response rate of 39%, with a median progression-free survival of 14 months and an OS of 22 months [80]. Those results compare favorably to previous studies, with a median OS of 14–15 months in unresectable iCCA populations treated with RE only [76,96]. Notably in the aforementioned Phase 2 study, with 22%, a significant number of patients were downstaged to resection. These patient groups did not achieve the median relapse-free survival at 46 months and the postsurgical 24-month OS rate was 88.9%. A Phase 3 trial is ongoing to corroborate these findings: the results of the SIRCCA trial (NCT02807181), evaluating RE followed by gem/cis vs. gem/cis alone as the first-line treatment of patients with unresectable iCCA, is expected to be released next year. The conversion to resectability using RE has also been reported in other studies [77,87,90]. In a study by Bourien et al., those patients had an OS of 51.9 months [77].

Frequent factors associated with the outcome in various studies were tumor size/tumor burden, response after RE, and performance status. In a study of 21 therapy-naïve, unresectable iCCA patients, Reimer et al. reported excellent results for patients with a low hepatic tumor burden (<25%), with an OS of 37.5 months [89]. Patients with a higher tumor burden achieved an OS of 15 months. Levillain et al. as well as Bourien et al. demonstrated that the efficacy of RE in patients with unresectable, relapsed and/or chemo-refractory iCCA was highly dependent on the delivered tumor irradiation dose [77,85]. Other RE studies in different tumor entities confirm the distinct correlation between survival and the absorbed tumor dose [97,98]. Variations in the response rates after RE indicate a medical need for optimizing dose prescriptions [99]. Preferably, an increase in the dose should come with no change of the safety profile regarding liver failure, as some studies indicate that liver toxicity remains a significant adverse factor, negatively influencing the outcome after intra-arterial treatment in iCCA [72,77,82]. Interestingly, it has been observed in many studies that the presence of a limited extrahepatic disease did not affect survival, underscoring that local tumor control and concomitant maintenance of liver function is crucial for the outcome [20,61]. However, a selection bias by the local investigators must be assumed here as the feasibility of RE in borderline cases within a metastatic stage of disease is often individually decided, and the exact extent of metastases is usually not specified more precisely in the analyses. Compared with the aforementioned treatment techniques, abscesses and bile duct complications are very rare after RE [62].

Table 1 gives an overview of the mentioned studies.

## 5. Conclusions

Studies on interventional radiologic therapies for iCCA have shown encouraging results in selected patients, so that these treatment options will gain increasing attention, especially given the limited options for systemic therapy. Patients with unresectable or recurrent iCCA seem to benefit from hepatic tumor control provided by local or locoregional therapies, even with the presence of extrahepatic spread. Reports of secondary resectability after trans-arterial treatment underline their efficacy and may provide a new perspective in the treatment management of unresectable iCCA. Furthermore, the value of systemic or targeted adjuvant therapy after local treatment also remains unclear, as there is no evidence in this setting. In contrast, recent studies suggest that adjuvant oral chemotherapy after resection may be beneficial [100,101,102]. Such therapeutic approaches could be used in individualized treatment concepts, but these data need to be validated in larger prospective trials. Meanwhile, interdisciplinary boards should discuss the sequence of therapy depending on the patient’s performance status and comorbidities, the location of the tumor, and the patient’s request, as well as the available resources. The toxicity profile of the different therapy forms is also an important influencing factor to consider, as the aim should not be to maximize, but to optimize therapy. Considering the available data and the own experience at our center, we believe that future prospective study formats should focus on complementing systemic therapies by classes of interventions (“toolbox”), rather than specific techniques to provide an appropriate therapy concept for local tumor control, depending on the particular circumstances such as tumor size/extent and location, and functional liver remnant, as well as patient-specific factors.

## Figures and Tables

**Table 1:** Overview of mentioned studies.

	Author, Year	Patients (*n*)	Extrahepatic Disease (%)	Prior Chemotherapy (%)	Prior Liver Directed Therapy (%)	Median Overall Survival from Treatment (Months)
**Thermal ablation**						
	Fu, 2011	12	8	N/A	100	30
	Giorgio, 2011	10	0	N/A	10	N/A
	Kim, 2011	13	8	0	0	39
	Kim, 2011	20	0	N/A	100	27
	Yu, 2011	15	40	7	13	10
	Fu, 2012	17	41	N/A	59	33
	Haidu, 2012	11	27	9	55	60
	Xu, 2012	18	0	N/A	56	9
	Zhang, 2013	77	0	N/A	100	21.3
	Butros, 2014	7	0	N/A	86	39 (mean)
	Takahashi, 2018	20	0	64	76	24
	Zhang, 2018	107	0	N/A	56	28
	Xu, 2019	56	20	N/A	100	31
	Brandi, 2020	29	0	0	0	28
	Díaz-Gonzàlez, 2020	27	0	0	0	31
**HDR-BT**						
	Kamphues, 2012	10	40	N/A	100	N/A
	Schnapauff, 2012	13	0	0	0	14
	Jonczyk, 2018	61	N/A	31	46	16 (<4 cm), 10 (≥4 cm)
**TACE**						
	Aliberti, 2008	11	N/A	N/A	N/A	13
	Kiefer, 2011	62	31	29	13	15
	Park, 2011	72	54	N/A	N/A	N/A
	Kuhlmann, 2012	26	42	19	8	12
	Vogl, 2012	115	N/A	N/A	N/A	13
	Aliberti, 2017	127	N/A	100	31	15
	Wright, 2018	41	56	44	10	15
	Goerg, 2019	21	0	57	57	13
	Ge, 2020	183	0	0	100	27 *
**RE**						
	Ibrahim, 2008	24	33	29	N/A	15
	Hoffmann,2012	33	24	79	54	10
	Mouli, 2013	46	35	35	15	N/A
	Rafi, 2013	19	58	100	21	12
	Camacho, 2014	21	N/A	100	48	16
	Filippi, 2015	17	76.5	88.2	23.5	15
	Mosconi, 2016	23	8.7	52	83	18
	Soydal, 2016	16	31	56	N/A	10
	Shaker, 2017	17	35	29	24	N/A
	Bourien, 2018	64	16	44	23	16
	Gangi, 2018	85	42	72	21	12
	Reimer, 2018	21	14	0	0	15
	Levillain, 2019	58	N/A	N/A	52	10
	White, 2019	61	41	92	28	9
	Bargellini, 2020	81	25	57	40	15
	Buettner, 2020	115	41	79	15	11
	Edeline, 2020	41	N/A	0	12	22
	Köhler, 2020	46	30	61	30	10
	Paprottka, 2021	73	51	71	38	12

* not stated if estimated from first diagnosis or first treatment. HDR-BT: high-dose-rate brachytherapy; N/A: not available; TACE: transarterial adioembolization; RE: adioembolization.
